# Biodegradable biomatrices and bridging the injured spinal cord: the corticospinal tract as a proof of principle

**DOI:** 10.1007/s00441-012-1352-5

**Published:** 2012-03-14

**Authors:** Elbert A. J. Joosten

**Affiliations:** Department of Anesthesiology, Pain Management and Research Center, Maastricht University Medical Hospital, Maastricht, The Netherlands

**Keywords:** Corticospinal tract, Spinal cord Injury, Biomaterials, Cell transplant, Regeneration

## Abstract

Important advances in the development of smart biodegradable implants for axonal regeneration after spinal cord injury have recently been reported. These advances are evaluated in this review with special emphasis on the regeneration of the corticospinal tract. The corticospinal tract is often considered the ultimate challenge in demonstrating whether a repair strategy has been successful in the regeneration of the injured mammalian spinal cord. The extensive know-how of factors and cells involved in the development of the corticospinal tract, and the advances made in material science and tissue engineering technology, have provided the foundations for the optimization of the biomatrices needed for repair. Based on the findings summarized in this review, the future development of smart biodegradable bridges for CST regrowth and regeneration in the injured spinal cord is discussed.

## Introduction

### Why is the corticospinal tract a proof of principle?

The corticospinal tract (CST) is considered the ultimate challenge to demonstrate if a repair strategy is succesfull in regeneration of the injured mammalian spinal cord. It all started from the observations made after the use of peripheral nerve grafts into the lesioned spinal cord: a significant regrowth of CNS axons was noted, although various population of axons tracts including the CST did not respond (David and Aguayo 1981; Richardson et al. 1980, 1984). In line with this, the CST is often considered to be the most difficult tract to regenerate, because the distance between the injury of CST axons in the lesioned spinal cord and the location of the cells of origin in the sensorimotor cortex exceeds that of every other tract in the nervous system. From these observations, it became and still is, a major scientific challenge to develop a strategy to enhance and stimulate regeneration of injured CST fibers in the lesioned spinal cord. Whereas the CST is easy to anterogradely label (the cells of origin are located in the cerebral cortex and thus accessible), tract-tracing and identification of the CST fibers is relatively simple. Furthermore, the fact that the CST develops postnatally in the mammalian spinal cord resulted in an extensive knowledge about factors and molecules involved in the outgrowth of this tract and this might be extremely helpful in the design of optimal repair strategies.

### Repair of injured spinal cord and regeneration of corticospinal tract

Repair of the injured mammalian central nervous system (CNS) and, in particular, the spinal cord has already been a major challenge for neuroscientists for many decades. The complexity of the spinal cord with its many ascending and descending fiber tracts, the numerous spinal cell populations, the inter-connectivity between various levels of the spinal cord combined with the characteristic response after an injury in the adult, as well as the variability in the location and impact of the lesion in the clinical situation, makes the development of appropriate repair strategies very complex and difficult. Furthermore, in most cases of human SCI, there is a significant loss of spinal cord tissue and cavity formation is an important obstacle, impeding axonal regeneration (McDonald and Sadowsky 2002). Hence, crucial within the development of repair strategies for regeneration of injured fibers is the bridging of the lesion, including both the ingrowth of injured fibers into the graft (or bridge) but certainly also further elongation and regrowth of axons out of the graft into the host tissue. In this context, it is important to understand that regeneration of injured nerve fibers is a complex of sequences in time and place. In general, regeneration or repair of the primary damage after lesioning of fibers implies the achievement of several steps (Joosten 1997): (1) directed regrowth of injured axons within and around the lesion-area; (2) elongation of the ascending and descending axons into rostral and caudal spinal cord areas, respectively; (3) specific target finding; (4) neo-synaptogenesis; and (5) remyelination of the regrown axons. Regeneration finally results in the re-establishment of functional synaptic contacts. In contrast, regrowth is defined as the initial aspects of the response of the injured fibers: i.e., sprouting and elongation. In this review, the response of injured corticospinal (CST) fibers is discussed in view of the sprouting and elongation into implanted biodegradable structures, which act as a bridge for these fibers in order to overcome the lesion area.

### Biomatrices and bridging materials

From a clinical point of view, the limited access to autologous donor material and the immunological reactions including allograft rejection have prompted the search for artificial biomaterials that may be implanted as bridges into the lesioned spinal cord. Bridging materials should ideally have a structure that is easily modifiable, absorbable and immunologically inert, whereas at the same time they can serve as a scaffold for matrix molecules and cellular implants (Novikova et al. 2003). Although not absolutely necessary, another major quality of the ideal matrix is *biodegradability*, which needs to be timed and related to the establishment of significant plasticity of the injured CNS and in particular related to regrowth of the injured CST fibers. Hence, among biosynthetic implants for tissue regeneration after SCI, both “Biodegradable natural polymers” and “Biodegradable synthetic implants” are discussed with special emphasis on the regrowth of injured (CST) fibers. For understanding the selection and design of the biomatrices or, often, so-called axon growth conduits, which are needed for bridging the injury site, basically two aspects are important: on the one hand, biomaterial technology and on the other hand, detailed knowledge of the factors and molecules needed for regrowth of the injured CST. Details on molecules, factors and/or cells needed for regrowth are often collected from studies on the development of an individual CNS tract, as it has been shown that each CNS tract needs axon tract-specific requirements for development and regeneration (reviewed by Deumens et al. 2005 for the descending axon tracts in rat spinal cord). It is intriguing to see and understand how outgrowing CST fibers enter the spinal cord and are able to find their appropriate target cell during development (see “Development of the corticospinal tract”). The rat CST is often used as a model in developmental studies (Stanfield 1992; Joosten and Bar 1999; Canty and Murphy 2008) and this has led to an extensive knowledge about the factors and molecules involved. Hence, intrinsic and extrinsic factors involved in the development of the CST have provided the basis for many studies on CST regrowth after spinal injury in the adult mammalian cord.

As mentioned before, another important aspect in the development and optimalization of biomatrices for repair and bridging of the lesion gap in the injured spinal cord (and in particular the CST) is related to material science and tissue engineering technology. Here, most know-how is based on studies on repair after peripheral nervous system (PNS) injury. Although autologuous peripheral nerve grafts still result in a superior regenerative performance or “gold standard” for peripheral nerve repair (Dellon and Mackinnon 1988), many investigations, including those using non-resorbable and resorbable materials for bridging the lesion, have been performed aiming at a further increase of the therapeutic success-rate (reviewed by Deumens et al. 2010). Also, the inclusion of biochemical signaling molecules or cellular components into the conduits or biomatrices has led to major advances in development of experimental repair strategies for injured PNS (reviewed by Deumens et al. 2010). This has finally led to the approval of one non-biodegradable nerve conduit and four biodegradable nerve conduits for clinical use by the US Food and Drug Administration (FDA) and from the European Union with a Conformite Europeenne (or CE) certification. The advances made in peripheral nerve repair strategies, in particular related to material sciences including matrix design and surface modification, as well as the creation of implantable devices that include recognition domains for cell attachment, the use of internally oriented matrices and the sustained release of neurotrophic factors, has now led to an advanced level of tissue engineering in the PNS field. This technology and application of advanced materials in the PNS field is far ahead of that in use and repair of injured CNS but at the same time may inspire tissue engineers to further develop even more “intelligent scaffolds” that are needed in the very complex situation of the injured spinal cord.

The present review will start with an update on our current knowledge on the factors involved in outgrowth of the developing CST. Then, the focus will shift to regeneration of injured CST after spinal cord injury in view of the strategies used to enhance this regrowth. The effect of local application of biodegradable bridging structures and biomatrices with or without supporting cells/molecules, in view of the impact on CST re-growth, is discussed. Finally, the findings are discussed with emphasis on future design of smart implants and regeneration of CST in human SCI.

As should be stressed, the impact of the secondary damage and the inflammatory response that accompanies neural injury and, related to that, the immunomodulatory strategies in order to enhance CST re-growth is not the main focus of this review. The effects of inflammation on axon regeneration in the mammalian spinal cord are highly complex (for review, see Benowitz and Popovich 2011): mainly destructive in the lesion center and often pro-regenerative in the area of secondary damage, which is located at a distance of the primary lesion. Although not included into this review, it should be noted that the inflammatory response as related to secondary damage after SCI may be closely interacting and direct the capacity and impact of bridging structures for repair. Inflammation has, for instance, been reported to interact and augment the ability of neurotrophins like Neurotrophin-3 (NT-3) to promote axon growth of CST fibers after spinal cord injury (Chen et al. 2008).

## Development of the corticospinal tract

### The outgrowing CST

**Fig. 1:**
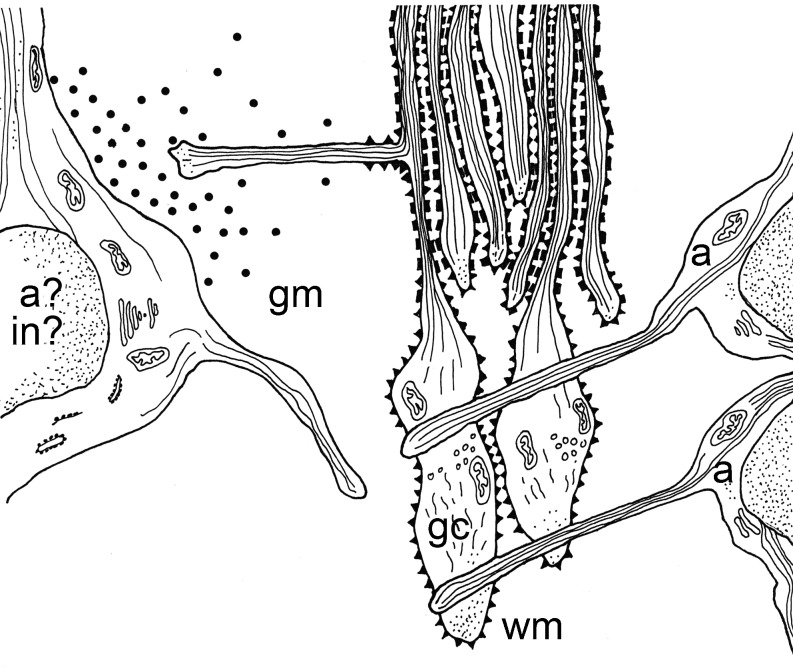
Schematic representation of outgrowth and guidance factors involved in the developing corticospinal tract in rat spinal cord. Vimentin immunoreactive astroglial cells (*a*) are situated in longitudinal tiers with their processes perpendicular to the outgrowing CST pioneer fibers and their growth cones (*gc*) (Joosten and Gribnau 1989a, b). The embryonic form of N-CAM is present on the growth cones of the pioneer CST fibers. The later-arriving CST fibers are guided by the cell adhesion molecule L1. During spinal gray matter target innervation, mainly through the formation of so-called back-branches, a tropic factor, probably NT-3, is released by either the CST target (inter)-neurons (*in*) or astroglial cells (*a*). Adapted from (Joosten 1997), with permission

The outgrowth of the corticospinal tract (CST) into the spinal cord of the rat is characterized by two phases, which both occur postnatally: a white matter tract formation on the one hand and the spinal gray matter target innervations on the other. Both phases are closely related and have been shown in various anterograde tract-tracing studies (Schreyer and Jones 1982; Joosten et al. 1987; Curfs et al. 1995; Joosten and Bar 1999). The outgrowth of main bulk of CST fibers is preceded by a relatively small number of pioneering axons with large growth cones (Gorgels et al. 1989). The majority of the CST axons follow these pioneering growth cones in tightly fasciculated bundles located in the ventralmost part of the dorsal funiculus (vDF) of the rat spinal cord. This staggered mode of outgrowth into the spinal white matter is shown in Fig. 1. After a waiting period (Donatelle 1977; Gribnau et al. 1986), the axons exit from the vDF by collateral branching in a rostro-caudal wave along the spinal cord (Kuang and Kalil 1991). Initially, these collaterals branch extensively in the gray matter, especially in the cervical and lumbar enlargements (Karimi-Abdolrezaee et al. 2002) where they finally transmit signals to muscles that control fore- and hindlimbs, respectively. This process of extensive collateral branching and arborization in the spinal gray is finally refined based on the connections made and mediated by the activity-dependent remodeling of the synaptic connections between the CST fibers and target interneurons (Ohno and Sakurai 2005). The interneurons have already then made contacts with the motoneurons in the ventral gray matter and the latter already have functional connections with the flexor and extensor muscles in fore- and hindlimbs. In view of functionality and the acquisition of voluntary movements, as this is the main function of the CST, it should be noted that learning the full complexity of locomotion is only possible if the CST is innervating not only the cervical but also the lumbar spinal cord. Then, finally, when the CST is fully grown, at the end of the third postnatal week, although myelination of fibers in the vDF may still take place (Gorgels 1990; Leenen et al.^1989^), the animals are able to execute very precise movements of the different muscle groups in particular in the forelimbs. This is therefore why the grasping test, which depends on the fine movements and interaction between forelimb digits, is considered a behavioral outcome of functionality of the CST in rats (Stackhouse et al. 2008).

Whereas the outgrowth of the dorsal and main component of the CST is described in detail and based on various tract-tracing and electron-microscopic studies, the development of the relatively small ipsi-lateral uncrossed CST component located in the ventral funiculus has received much less attention. The ventral CST penetrates the spinal cord white matter up to lumbar levels (Joosten et al. 1992) and finally forms branches into the spinal gray (Brosamle and Schwab 1997; Gianino et al. 1999). In view of the regeneration and regrowth of the CST after spinal cord injury, the presence of this ventral CST component is often neglected. It does not need further comment that lesion models focused at the dorsal part of the spinal cord in which in particular (and only) the vDF is transected or compressed, the fact that the ipsilateral ventral CST is still intact may lead to incorrect conclusions on the regrowth or regenerative response of lesioned CST fibers.

### Factors involved in CST outgrowth during development

The developing CNS is characterized by an overproduction of neurons, dendrites and axons in order to ensure that each target cell structure eventually receives an adequate input. This overproduction is followed by a process of elimination that is needed to match the size of the innervating population of cells and the capacity of the target: “system matching”. In the context of this phenomenon, a phase of naturally occurring cell death was observed in many parts of the CNS (Hamburger and Oppenheim 1982). However, cell death is virtually absent in the layer V of the cortex, where the large pyramidal cells are located, which are the cells of origin of the CST (Armand 1982, among others). Not cell death but the overproduction and subsequent elimination of long CST projections plays an important role in CST development as elegantly shown in a double-labeling retrograde experiment (Stanfield 1982). In the context of regeneration of the CST after spinal injury, it has long been a controversy whether the cells of origin of the CST die or not. It has even been claimed that neurotrophic factors like neurotrophin-3 or brain-derived neurotrophic factor (BDNF) are needed for survival of adult axotomized CST neurons and applied to the cortex, because when a cell body dies, the axon dies as well (Carlson et al. 2000). Hence, in order to induce or establish repair after spinal cord injury, both an intervention aimed at the survival of the cells of origin and at the same time those aimed at enhancing the axon regeneration at the lesion site are needed. However, a recent detailed imaging and histological analysis on Wallerian degeneration and axon number in the medullary pyramids of adult rats after acute SCI showed that SCI caused no cell death of CST cell bodies in the cortex (Nielson et al. 2010). The latter implicates that therapeutic strategies aimed at promoting CST axon regeneration in the spinal cord do not require a separate intervention aimed at protection of cellular death of the cells or origin (Nielson et al. 2010).

The developing mammalian CST is characterized by a staggered mode of outgrowth (Schreyer and Jones 1982; Gribnau et al. 1986). A main wave of later-arriving axons is preceded by a number of pathfinding axons, which are characterized by dilatations at their distal ends: the growth cone (Joosten et al 1992; for review, see Joosten and Bar 1999). These pioneer growth cones are 10 µm wide and 25 µm long and include fusiform, clavate, lamellipodial and filopodial morphologies, whereas the later-arriving axons are relatively straight and about 0.5 µm in diameter (Gorgels 1991; Joosten et al. 1992; Joosten and Bar 1999). The pioneer fibers must receive information from the environment, which directs their direction and speed of growth, whereas those axons that follow are guided by means of fasciculation with the pioneers (for review, see Joosten 1997) (Fig. 1).

The environment of the DF that the pioneer CST will face upon arrival is predominantly made up of interweaving processes of immature glial cells, with some neuronal processes from the surrounding gray matter (Schreyer and Jones 1982). From a molecular point of view, there is evidence that Eph-ephrin signaling is involved in the generation of the DF, as, for instance, Ephrin A4 and its ligand ephrin-B3 are expressed throughout the embryonic DF (Greferath et al. 2002; Dottori et al. 1998; Kullander et al. 2001). Detailed immunohistological studies have shown that in particular astroglial cells are pivotal in guiding the pioneer CST growth cones: vimentin-immunoreactive immature astroglial cells are situated in longitudinal tiers with their processes perpendicular to the outgrowing CST growth cones (Joosten and Gribnau 1989a) and, moreover, junctional specializations between these growth cones and the glial processes have been documented (Fig. 1) (Joosten and Gribnau 1989a; Gorgels 1991). In addition to the glial cell-mediated mechanical way of routing, molecular cues have been identified to be important in this guidance and glial cell-mediated interaction: the presence of the embryonic form [highly poly-sialylated (PSA)] of the cell adhesion molecule [neural cell adhesion molecule (N-CAM)] on the CST growth cones in spinal white matter strongly suggests involvement of this molecule in the guidance of the CST fibers (Joosten 1994; Joosten et al. 1996; Daston et al. 1996). The bulk of later-arriving fasciculating axons is guided through the differential adhesiveness by the cell adhesion molecule L1 (Fig. 1) (Joosten and Gribnau 1989b; Joosten et al. 1990). The insulin growth factor-1 (IGF-1) has also been implicated in axonal elongation through the vDF (Ozdinler and Macklis 2006) as shown through the in vivo blockade of the IGF-1 receptor using antibodies; this significantly reduced the CST axon outgrowth in the spinal white matter (Ozdinler and Macklis 2006). Another interesting finding is the role of Wnt proteins in CST growth cone guidance: both Wnt1 and Wnt5a were shown to repel CST axons due to a repulsive signaling mediated through the Ryk receptor (Liu et al. 2005); in fact, CST axons are repelled from areas of high Wnt expression and as a result prevent the CST axons from exiting the DF.

After a waiting period of several days, some CST fibers change their direction, veering away from the tract and directly entering the adjacent spinal gray matter (Joosten et al. 1994; Joosten and Bar 1999). Nevertheless, CST target innervation mainly occurs by formation of collateral branches (O’Leary and Terashima 1988; Joosten et al. 1994), which occurs at defined locations or so-called ”decision-points” (O’Leary and Terashima 1988; Kuang et al. 1994; Joosten et al. 1994). Most of the collateral branching occurs at cervical and lumbar regions (enlargements), which are the two major CST termination zones in the spinal cord. The spinal target innervation of the CST fibers are thought to be mediated through gradients of diffusible neurotropic molecules, which are either released by neuronal or by glial target cells located in the target areas, as shown in vitro collagen co-culture studies (Fig. 1) (Heffner et al. 1990; Joosten et al. 1991, 1993, 1994; Sato et al. 1994). A major candidate neurotrophin is the NT-3 as its receptor tyrosine kinase -3 (trk-3) is present on the cells of origin of the CST (Frisen et al. 1993). Levels of NT-3 mRNA in spinal motor neurons (i.e., CST target cells) are significantly increased at early postnatal time points as compared to the adult spinal cord where NT-3 mRNA cannot be detected (Ernfors and Persson 1991). In addition to the neurotrop(h)ic chemotactic guidance of outgrowing CST fibers, contact-mediated guidance is also thought to be involved in the CST target innervations: the CST fibers discriminate target tissues by direct contact with the gray matter, as shown in in vitro co-culture experiments (Kuang and Kalil 1991).

Furthermore, extracellular matrix (ECM) molecules and myelin-associated proteins may act as outgrowth inhibitory molecules and be implicated in the outgrowth and restriction of CST pioneer fibers to leave the DF during their descent (Schwab and Schnell 1991). High levels of chondroitin sulfate proteoglycan is observed in the area surrounding the DF, particularly at the time of arrival of the leading pioneer CST fibers (Hsu et al. 2005), suggesting the formation of a barrier to the exit of the CST fibers in the DF white matter.

#### In summary

The various diffusible as well as contact-mediated guidance cues, which might either be permissive or repellent for outgrowth of CST fibers in spinal white matter, have been described and are identified. The three-dimensional structure and arrangement of immature outgrowth promoting cells or glial cells including outgrowth permissive cell adhesion molecules (N-CAM/ L1), in the environment of the vDF upon and during the arrival of the first CST pioneer fibers, is important for guidance. For correct target innervation (back-branching of fibers) and contact formation of the CST fibers, both outgrowth stimulating as well as outgrowth inhibitory molecules including various CAMs, EphA4, growth associated protein 43 (GAP-43), chondroitin sulphate proteoglycans and identified (NT-3) (or as yet unidentified) neurotrophins are needed. The know-how on cells and molecules involved in CST outgrowth during development is important in the design and creation of optimal bridging structures, which are used to enhance and direct the regrowth of injured CST fibers in adult mammalian spinal cord.

## Biomatrices and bridging structures for CST regrowth after SCI

A damaged peripheral nerve is able to regrow its axons through the distal stump, mainly via the bands of Bungner that are composed of tubes of basal lamina enclosing Schwann cells (SC). A prerequisite for this regrowth of injured PNS axons is the presence of a physical continuity. Hence, in large peripheral lesion gaps, many bridging materials have been tested to allow and stimulate the regrowth of injured fibers. In contrast to the injured PNS axons, those of the CNS fail to regenerate, although local sprouting can occur. The pioneering work of Ramon Y Cajal (1928) indicated that, if the environment is suitable, axons from injured central neurons are able to regrow. With the development of advanced anatomical tract tracing techniques, the regrowth of CNS axons into the peripheral nerve grafts was convincingly demonstrated (David and Aguayo 1981). Although this peripheral graft allows axons of various CNS populations to regrow, the PN-graft seems to be rather selective for certain categories of CNS axons. In this respect, it is important to know that injured CST axons do not respond to PN-grafting of the lesioned spinal cord (Richardson et al. 1980, 1984). These findings clearly initiated a large body of research on use and development of biodegradable bridging materials (natural polymers or synthetic in origin) with or without supporting cells/molecules in order to stimulate the regrowth of injured CST axons.

### Biodegradable natural polymers

A variety of biodegradable natural polymers have been used as implants and cell carriers for repair after SCI. Among them collagen type I and alginate have received most attention.

#### Collagen

Developmental studies have shown that environmental factors such as extracellular matrix molecules (ECM) components [e.g., laminin, fibronectin, heparin sulphate, collagen and/or cell adhesion moelcuels (CAMs)] are transiently expressed during periods of axonal elongation (Botz et al. 1990), including the CST (for review, see Canty and Murphy 2008). As a biodegradable material, the ECM component collagen is promising due to its abundant sources, good plasticity and biocompatibility (Lee et al. 2001). Axonal regrowth is reported after collagen is grafted parallel to the axis of the transected spinal cord (Yoshii et al. 2004). Besides a direct growth-promoting effect (Harris et al. 1985), collagen implantation into the lesion gap of the injured spinal cord may also be important in stimulating the regrowth of lesioned CST fibers by functioning as a scaffolding structure. Collagen (type 1) is composed of a cross-linked lattice network of fibers and fibrils with abundant inter-fibrillary spaces (Gross and Kirk 1958) and in vitro studies have shown the biocompatibility of collagen hydrogels for neural tissue (Harris et al. 1985). Implantation of a collagen gel into the lesioned adult rat spinal cord may result in ingrowth of injured fibers (Marchand and Woerly 1990) including CST fibers (Joosten et al. 1995a). The latter is, however, considerably affected by the method of application (Joosten et al. 1995a): whereas the application of a fluid collagen graft, which self-assembles in situ, results in the invasion of astroglial cells and at the same time (4 weeks post-implantation) ingrowth of a significant number of anterogradely labeled CST fibers, the implantation of an already self-assembled gel resulted in the absence of any ingrowing CST fibers (Joosten et al. 1995a). It is suggested that the optimal integration of the fluid collagen matrix and re-establishment of a physical continuity to the transected spinal cord may account for the ingrowth of CST fibers (Marchand and Woerly 1990; Joosten et al. 1995a). However, no regrowth of injured CST fibers caudal to the graft was noted in either of the collagen implants, which either suggest that the caudal stump of the adult spinal cord is still non-permissive or that the regrowth of the CST fibers in the fluid collagen graft, despite the presence of invading astroglial cells, lacks the presence of directed guidance. Despite the fact that CST axons grow into the collagen matrix placed between the stumps of the partially transected spinal cord, the matrix became denatured after 2–3 months and, as stated by Marchand and collegues, the invasion of injured axons into the matrix is then not fully developed (Marchand et al. 1993). The collagen biomatrix becomes highly disorganized by the massive infiltration of endogenously synthesized connective tissue. In this, the artificial matrix is not able to withstand the tendency of the newly produced (connective) collagen to form aggregates, thus leading to its denaturation. The stability of the biomatrix could be improved by co-prepitation of the collagen with chondroitin-6-sulfate or chemically cross-linked with carbodiimide (CD) (Marchand et al. 1993). The CD collagen cross-linking treatment improved the stability of the collagen type I. The stabilized collagen type 1 biomatrix modified the normal host scarring process, which then favored axonal regeneration (Marchand et al. 1993). Unfortunately, analysis of CST regrowth was not included in this study.

The solid collagen implant may have certain advantages over the fluid injection of collagen with respect to manipulatibility/controllability, which is particularly important if the collagen acts as a vehicle for the local application of cells and/or neurotrophic factors (Joosten et al. 1995a). Immature astroglial cells embedded in a collagen gel were implanted into the lesioned spinal cord of the rat, thereby mimicking to some extent the developmental situation (compare and see “Development of the corticospinal tract”; Joosten and Gribnau 1989a). In this context, it is important to note that astroglial cells derived for neonatal rat cortex was used. Although it has been documented that target-derived astroglial cells preferentially stimulate axon length of defined neuron populations (Rousselet et al. 1990, among others), in vitro experiments have shown that CST axon growth is not influenced by the anatomical origin (cortex vs. spinal cord) of the astrocytes (Dijkstra et al. 1999). Although the use of collagen as a vehicle to transplant neonatal cortical astroglial cells into the lesioned spinal cord of the adult rat resulted in a significant increase in the number of ingrowing neurofilament positive fibers, including anterogradely labeled CST axons, no improvements were noted in locomotor behavior (Joosten et al. 2004). The close contact between the transplanted immature cortical astroglial cells and the regrowing CST fibers in the collagen matrix suggest a contact-mediated guidance phenomenon, although it might also be possible that the enhanced ingrowth is due to the release of neurotrophins like Neurotrophin-3 (NT-3), a factor known to be released by neonatal astrocytes (Althaus and Richter-Landsberg 2000). On the other hand, no re-entry of regrowing CST axons was observed into the host tissue after the implantation of immature cortical astroglial-collagen grafts (Joosten et al. 2004). This indicated that, in addition to the collagen-astroglial matrices, additonal stimulation will be necessary. In this respect, the olfactory ensheating cells (OECs) were very promising candidates. OECs are a unique population of peripheral glial cells sharing both Schwann cell and at the same time astrocyte characteristics (Barnett and Chang 2004; Lakatos et al. 2000). Moreover, the transplantation of OEC’s into very restricted spinal cord lesions resulted in an extensive elongation of injured CST fibers into the caudal host tissue and this is associated with functional restoration of specific motor tasks (Li et al. 1997; Ramon-Cueto et al. 1998, 2000). After injection of the OECs into very small lesions in the spinal cord, these cells have been reported to migrate into the CNS, thereby promoting the re-entry of regenerating axons (Ramon-Cueto and Nieto-Sampedro 1992). Although the injection of OECs showed promising effects on the repair of injured CST axons across small and chronic lesion gaps (Li et al. 1997, 1998; Ramon-Cueto et al.^1998^, ^2000^; Keyvan-Fouladi et al. 2003), the effects of injecting OECs directly into a large lesion gap of the injured spinal cord were very limited (Li et al. 2004) . In order to improve this outcome, the use of collagen as a vehicle has been considered and the behavior and characteristics of OEC on 2-D as well as 3D collagen matrices have been studied (Wang et al. 2006). These in vitro studies suggested that 3-D collagen scaffolds provide suitable environments for the OECs to maintain their morphology as well as several important functional phenotypes (including the production of neurotrophic factors) and all these could be helpful for the effective treatment of SCI. However, no in vivo follow up studies have been reported that show the possible beneficial effect of these 3D collagen OEC scaffolds and regeneration of CST fibers after SCI.

It is interesting to note that the response of CST axons on the OEC implanted cells, at least in the smaller spinal lesions, is probably mediated by the release of the diffusible neurotrophic molecule NT-3 (Ramon-Cueto et al. 1998), a molecule known to be important during development and guidance of the outgrowing CST (see “Development of the corticospinal tract”).

As the release of neurotrophins by OECs transplanted into the lesioned spinal cord may be important in the stimulation of the regrowth of injured axons, including the CST fibers, one could hypothesize that the local delivery of neurotrophic molecules by itself is sufficient for CST regrowth. Here, collagen may also act as a vehicle for local application and thus various experiments have been designed where either known neurotrophic factors like BDNF or NT-3, or extracts derived from postnatal spinal target tissue known to release tropic substances that attract the CST fibers, (see “Development of the corticospinal tract”) were used.

In view of the idea that regeneration is based on a repetition of the development, the use of target-derived extracts is challenging. Based on the in vitro findings that chemotropic factors are released by the spinal gray target and specifically direct and attract outgrowing CST fibers (Heffner et al. 1990; Joosten et al. 1991, 1994; Sato et al. 1994; see “Development of the corticospinal tract”), it was hypothesized that timed postnatal cervical or lumbar spinal cord gray matter extracts may contain the appropriate trop(h)ic factor to stimulate CST re-growth into the collagen matrix, after SCI. Indeed, transplantation of the extracts using collagen type I as a vehicle into the T8/T10 dorsally hemisected spinal cord of an adult rat resulted in a very strong increase in the re-growth of injured CST fibers, although no regenerating CST fibers were noted in the caudal host tissue (Joosten et al. 1995b). Several lines of evidence suggested that NT-3 (or BDNF) might be the neurotrophic factor involved. And, indeed, similar results were reported after the local application of NT-3 into the lesioned spinal cord of an adult rat using collagen as a vehicle: collagen containing NT-3 implantation resulted in a directed regrowth of the injured CST fibers towards the source of the NT-3 (Houweling et al. 1998). Also, transplants of modified NT-3 secreting fibroblasts embedded in collagen type I increased the regrowth of severed CST axons; however, surprisingly not into the graft (Grill et al. 1997). The avoidance of the regrowing CST axons into the collagen graft area containing the NT-3 secreting fibroblasts was compensated by the presence of regrowing CST fibers underneath the T7 dorsal hemisection lesion sites (Grill et al. 1997). Interestingly, the rats with NT-3 containing collagen implants as well as those containing the NT-3 secreting fibroblasts showed a similar improvement at the functional level as measured with the gridwalk test (Grill et al. 1997; Houweling et al. 1998). As the absence of CST regeneration caudal to the lesion results in improvements in the gridwalk test, it was concluded that this behavioral test is not sensitive enough for detection of CST-mediated functional regeneration (Houweling et al. 1998). For correct analysis of CST regeneration after mid-thoracic SCI a more sensitive functional test is needed.

CST regrowth has been studied based on local application of various other neurotrophic factors. BDNF and NGF both secreted by genetically modified fibroblasts were applied to the lesioned spinal cord using collagen type I as a vehicle and bridging substance (Blesch et al. 1999; Tuszynski et al. 1997). In particular, BDNF is an interesting candidate for stimulation of regrowth of injured CST fibers due to the fact that the functional receptor of BDNF, full length tyrosine kinase B (trkB), is present on the somata of CS neurons in the sensorimotor cortex (Lu et al. 2001). However, without exception, the local deliveries of neurotrophic factors using a collagen-gel as a vehicle and applied into the injured spinal cord did not result in regrowth of the CST fibers into areas caudal to the lesion (Blesch et al. 1999; Tuszynski et al. 1997; Joosten et al. 1995b; Houweling et al. 1998; Tobias et al. 2005).

##### In summary

**Fig. 2:**
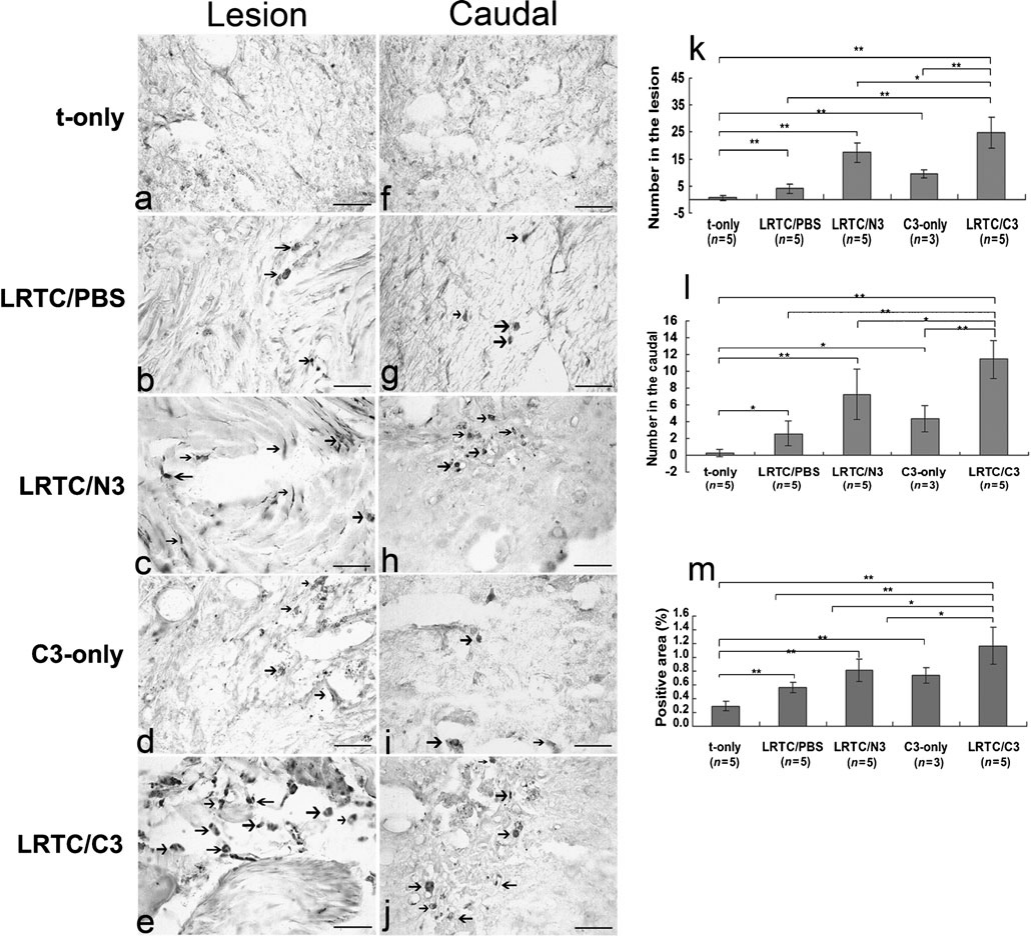
Detection and quantification of biotinylated dextran amine (BDA)-labeled corticospinal tract (CST) fibers. (**a–j**) Photomicrographs of the lumbar region of the spinal cord showing axons regenerating in the CST labeled with BDA in cross-sections. The sections were 400 μm from the border of the lesion and the caudal stump. There were differences in the appearance of the lesion and the caudal portion.* Arrows* indicate BDA-labeled CST fibers (*scale bars* 50 μm). The numbers of BDA-labeled fibers in the lesion (**k**) and the caudal stump (**l**) and the percentages of positive BDA-labeled fibers per section in the lesioned and caudal segments (**m**) are shown. There was a statistically significantly larger positive area in the LRTC/C3 group than in the other groups (*p < 0.05, **p < 0.01 as determined by the two-tailed Student's *t* test).* LRTC/PBS* linear rat-tail collagen/phosphate-buffered saline animals,* LRTC/N3* linear rat-tail collagen/neurotrophin-3 animals,* LRTC/C3* linear rat-tail collagen/collagen-binding neurotrophin-3 animals,* C3-only* collagen-binding neurotrophin-3 only animals,* t-only* transected-only animals). Adapted from Fan et al. (2010), with permission

The strategies described are based on local delivery of neurotrophins (either pure or produced by genetically modified fibroblasts) using collagen as a vehicle that is not sufficient for CST regrowth and re-establishment of functional connections. Although collagen can serve as a bridge to connect the rostral and caudal portions of the injured spinal cord (Joosten et al. 1995a), significant improvements need to be made before optimal regeneration of CST fibers can be established. In this respect, recent developments need to mentioned: the establishment of an increased binding ability of either NT-3 (Fan et al. 2010) or BDNF (Liang et al. 2010; Han et al. 2010) to collagen, based on the construction of recombinant fusion proteins; the development of recombinant collagen-binding NT3 (CBD-NT3), consisting in a collagen-binding domain (CBD) and native NT3; as well as the same time the formation of linear ordered collagen scaffolds that direct the outgrowing CST fibers, are all important improvements (Fan et al. 2010). Indeed, the use of linear ordered tail collagen scaffolds (LRTC) loaded with CBD-NT-3 was shown to promote anterogradely labeled CST regrowth into the matrix after transplantation into a complete spinal transection (Fig. 2) (Fan et al. 2010). However, here again, no regeneration of CST fibers was noted into the caudal host tissue (Fan et al. 2010), although the authors report at the same time functional recovery as assessed by use of the Basso-Beattie-Bresnahan (BBB) locomotor rating scale (Basso et al. 1995) or the gridwalk. As discussed before, neither the BBB nor the gridwalk tests are correlated to the functionality of the CST.

#### Alginate

Alginate is a naturally derived polysaccharide extracted from brown seaweed and composed of 1,4-linked B-D-mannuronate and 1,4-linked alpha-L-glucuronate. It forms a gel by cross-linkage with calcium ions, is immunologically inert- and not digested by mammalian cells. As the calcium ions gradually diffuse out of the gel, it slowly degrades and is excreted in the urine (Novikova et al. 2003). Although the commercially available alginate is thought to be cytotoxic, the development of a novel alginate sponge, cross-linked with covalent bonds, no longer showed inhibitory effects on cell proliferation in vitro. The use and implantation of freeze-dried alginate sponges into the lesion gap in completely transected spinal cord of young adult rats has been shown to stimulate the re-growth of myelinated and un-myelinated fibers into this hydrogel (Suzuki et al. 1999a, b; Kataoka et al. 2004). Regrowth of both ascending and descending axons that traversed the alginate-filled gap included the re-entrance of the host spinal cord and the formation of electrophysiological active neuronal connections with host neurons (Suzuki et al 2002). Regenerating axons grew 1–1.5 cm rostrally and 200–300 mm caudally from the lesion through the host spinal cord tissue. Twenty-one weeks after implantation, many regenerated axons from the CST were observed throughout the gap in six animals. Some HRP-labelled CST axons extended a further 200–300 mm through the white and gray matter caudal to the gap in four animals and the number of regenerated CST axons was much smaller than that of regenerated axons in the ascending pathway (Suzuki et al. 2002). The fact that re-growing CST axons were able to re-enter the host tissue after use of the alginate hydrogel sponge, as compared to the absence of re-entry of CST fibers after collagen implants (see “Collagen”) was suggested to be related to the fact that collagen is a component of the extracellular matrix that enhances proliferation of fibroblasts. The accumulation of fibroblasts in the spinal cord gap may act as an additional physical barrier. From this, it should be concluded that the alginate sponge has major advantages over the collagen-gel implants. However, the report that alginate freeze-dried sponges stimulate regrowth of CST fibers, including the re-entry into the host tissue, is at present limited to one publication (Suzuki et al. 2002).

The development of alginate hydrogels as potential cell carriers for neurotransplantation has been considered (Novikova et al. 2006; Tobias et al. 2001, 2005). Alginate hydrogels may act as carriers for extracellular matrix molecules and cell lines, stem cells and neurotrophic factors. In vitro studies have shown that alginate hydrogels transformed olfactory nerve ensheating cells (ONEC), Schwann cells (SC) and bone marrow-derived stem cells (BMSC) into atypical cells with spherical shape and decreased the metabolic activity of the cells (Novikova et al. 2006). The addition of the extracellular matrix should therefore be considered when engineering biosynthetic scaffolds on the basis of alginate hydrogels, because this does not result in (morphological and metabolic) changes of the transplanted cells (Novikova et al. 2006). On the other hand, in particular for clinical application, the encapsulation of cells is needed to provide a protective barrier against host immune cell interactions after grafting. The alginate capsules are coated with a poly-cation, such as poly-ornithin, which forms a size exclusion barrier that limits the size of substances that pass into and out of the gel, so that growth factors diffuse out but immune cells cannot target the foreign cells within the capsule (Tobias et al. 2001). Alginate-encapsulated BDNF-producing fibroblast grafts have been shown to result in both axonal regrowth, which seems to be rather undirected and at the same time recovery of function after spinal cord injury. However, anterograde tract tracing did not show any regeneration of rubrospinal axons in the recipients of alginate-encapsulated BDNF fibroblasts (Tobias et al. 2005). Hence, injured long descending tracts do not regrow in the presence of alginate hydrogels containing cells secreting neurotrophic factors. It is, however, discussed that the amounts of BDNF available from the alginate-encapsulated BDNF-producing fibroblasts is substantially less than those provided by much larger numbers of BDNF-producing fibroblasts that were injected into the lesion gap of an injured spinal cord. The latter approach was reported to result in regrowth of injured RST and CST fibers ( reviewed by Deumens et al 2005). Obviously a target for future research is the creation of alginate encapsulated neurotrophin-producing cells, which release substantially more of the neurotrophic factor. Nevertheless, it should be taken into account that this approach may result on the one hand in increased regrowth of descending fibers into the graft but on the other hand that this ingrowth is still undirected.

The most recent development in use of alginate-encapsulated neurotrophin-producing fibroblasts is the use of various cross-linking methods, which allow both promotion of regrowth of injured fibers and sculpting of the hydrogel into an optimal shape for bridging and guiding the outgrowing nerve fibers (Francis et al. 2011).

##### In summary

The use of alginate as a biomaterial for bridging the injured spinal cord may result in significant CST regrowth including the re-entry into host tissue, although this finding was limited to one publication. Hence, more research is needed to further analyze the effect of alginate in the repair and regeneration of injured CST. The use of aliginate hydrogels and aliginate capsules allows the transplantation of neurotrophin-producing cells (fibroblasts), which is a very promising approach in repair and bridging the injured spinal cord. Future research should be aimed at the creation and transplantation of aliginate-encapsulated cells producing substantially more of the neurotrophic factor.

### Biodegradable synthetic implants

Synthetic biodegradable implants tested for spinal cord repair include matrigel matrix, fibrin and fibronectin (mats) and poly (alpha-hydroxy acids).

#### Matrigel

Matrigel is a soluble basal membrane extract of the Engelbreth-Holm-Swarm tumor cell line. It forms a nonporous hydrogel at room temperature and the matrigel matrix contains laminin, collagen IV and heparin sulphate proteoglycan, as well as growth factors such as insulin growth factor-I (IGF-I). The implantation of Matrigel alone into the injured spinal cord does not stimulate regeneration of injured axons (Bunge 2002; Facchiano et al. 2002), an effect that is likely to be related to the inhibitory effects of collagen IV on axon growth (Hermanns et al. 2001), although the latter is not conclusive as immunohistochemical double staining revealed that penetrating neurofilament-positive regrowing axons and collagen IV deposits were co-localized at the lesion site in the initial stages of axonal sprouting (between 7 and 14 days postoperative) and were still present 1 and 2 months post-operatively (Joosten et al. 2000). Interestingly, collagen IV-deposits around cystic cavities, formed at the injury site 1 month post-lesion, were devoid of axons (Joosten et al. 2000). Despite the questionable role of collagen IV (as part of the Matrigel matrix) on axon outgrowth, Matrigel has been used as a bridge and for the local application of axon outgrowth stimulating factors and/or cells into the lesioned spinal cord.

Among those cells most often used for transplantation are the Schwann cells (SC) (Bunge 2002). The use of human SC grafts transplanted into the lesioned spinal cord immediately after injury resulted in the regrowth of sensory and propriospinal axons from the graft into the host, although only a small proportion (about 1%) of the fibers entering the human SC grafts showed evidence of regrowth from the graft into the host spinal cord (Guest et al. 1997b). Unfortunately, these SC grafts were characterized by the absence of ingrowing severed CST fibers (Guest and Bunge 1995; Chen et al. 1996; Xu et al. 1995b). A complicating factor is the survival of the transplanted SC after grafting. If SC were embedded in Matrigel and grafted within polyacrylonitrite/polyvinylchloride copolymer (PAN/PVC) guidance channels, increased cell survival was noted and at the same time the formation of a compact cable inside the channel (Guest et al. 1997a). Strictly speaking, the scaffold tested in these experiments (Guest et al. 1997a) is a composite conduit based on the combination of non-biodegradable rigid material (PAN/PVC) filled with a biodegradable hydrogel (matrigel).

Matrigel and local application of neurite outgrowth promoting (stem) cells, in particular Schwann cell implantation, have shown promise in overcoming many of the obstacles facing successful repair of the injured spinal cord including the successful survival of transplanted cell (or cell suspensions). The implantation of Schwann cells as cell suspensions with in situ gelling Matrigel but also with collagen as a vehicle, after spinal cord contusion significantly enhances long-term cell survival but not proliferation, as well as improvement of graft vascularization and the degree of axonal in-growth over the standard implantation vehicle (Patel et al. 2010). The use of Matrigel to suspend cells prior to implantation is an important consideration for achieving improved survival and effectiveness of cellular therapies for future clinical application. Although use of SC/Matrigel-filled PAN-PVC guidance channels (after a *complete transection*) did result in an improved cell survival, the additional use of fibrin glue and acidic fibroblast growth factor (aFGF) was needed to demonstrate regrowth of injured anterogradely labeled CST fibers (Guest et al. 1997a). Grafting the space between the SC/Matrigel-filled PAN/PVC bridges and the spinal cord stumps with aFGF and fibrin glue, where the stumps of the spinal cord were inserted 1 mm into each end of the channel, resulted in some regrowth of injured CST fibers into the graft and at the same time a reduced die-back of CST fibers (Guest et al. 1997a). Obviously, in this transplantation paradigm, the use of fibrin scaffolding is needed for regrowth of injured CST fibers [for more on the role of fibronectin and regrowth of CST, see “Fibrin and Fibronectin (mats)”]. At the same time, it is worth mentioning that the type of lesion model also considerably affects the outcome of the grafting procedure: if Matrigel-embedded SCs in PAN/PVC guidance channels were placed into *partially transected* spinal cords of the adult rat, the neurons from a number of brainstem regions extended not only their fibers into the graft but grew into the distal spinal cord and formed bouton-like structures (Xu et al. 1999). The response of the brainstem neurons after a partial transection of the cord may result, at least in part, from the restoration of cerebrospinal fluid circulation and relatively more stable cord-graft interfaces due to the more limited laminectomy (Bunge 2001). Additional infusion of neurotrophins BDNF and/or NT-3 into the distal cord parenchyma further promoted axonal regrowth into the distal host spinal cord (Xu et al. 1995a; Bamber et al. 2001). Although NT-3 is specifically related to the guidance and outgrowth of CST fibers (see “Development of the corticospinal tract”), the identification of the regrowing fibers and thus the CST, is a prime candidate to respond to this two-phase strategy, as the fiber analysis was restricted to anterogradely labeled central axons due to the fact that the tracer was only injected into the spinal cord 3 mm rostral to the graft. Based on this procedure, the exact origin of the regrowing central fibers is not clear but at the same time does not exclude the possibility that regrowing CST fibers are involved (Bamber et al 2001).

Matrigel is not only used as a vehicle to introduce SCs into the lesion area but also for local delivery of stem cells: human bone marrow stromal cell-derived Schwann cells (hBMSC-SC) (Kamada et al. 2011) or human umbilical cord blood (HUCB) enriched with CD34-positive cells (Nishio et al. 2006). Human bone marrow stromal cells (hBMSC) were cultured from bone marrow of adult human patients and induced into Schwann cells (hBMSC-SC) in vitro.

Nine days after injury, a mixture of Matrigel and hBMSC-SC (hBMSC-SC group) was injected into the lesioned site. Five weeks after transplantation, the application of Matrigel and hBMSC-SC resulted in a reduced cystic cavitation and at the same time promotion of the functional recovery (Kamada et al. 2011). Histologically, the number of tyrosine hydroxylase- or serotonin-positive fibers was significantly larger at the lesion epicenter and caudal level in the hBMSC-SC group than in the control group. However, no CST tract tracing was included and thus the effect of this transplantation approach on CST regrowth still needs to be determined.

As graft vascularization may be closely related to the degree of axonal in-growth, further increase of blood vessel formation or angiogenesis (and thus the survival of the transplanted cells) has been studied using Matrigel-containing vascular endothelial growth factor (VEGF). With the use of Matrigel as a vehicle, exogenous vascular endothelial growth factor (VEGF165), either as recombinant protein alone or combined with an adenovirus coding for VEGF165, was applied into the lesion gap of the transected spinal cord (Facchiano et al. 2002). The rationale behind this VEGF165 application was that the absence of regeneration of CST fibers after injury is related to the deficiency of both blood flow and growth factors. Hence, the exogenous addition of vascular endothelial growth factor (VEGF165) to the transected spinal cord, either as recombinant protein alone or combined with an adenovirus coding for VEGF165, was suggested to result in an increased blood vessel formation (angiogenesis), which might permit the regeneration of the CST fibers. The local application of Matrigel-containing recombinant VEGF165 or Ad.CMV.VEGF165 resulted in a reduced retrograde degeneration of CST axons as compared to rats treated with Matrigel alone or Matrigel plus Ad.CMV.LACZ. (control adenovirus). Furthermore, in rats treated with recombinant VEGF165 alone, or combined with Ad.CMV.VEGF165, a few anterogradely HRP-labeled CST axons, which were not detectable in control rats, were seen distal to the spinal cord injury, indicating some CST regrowth across the injured area (Facchiano et al. 2002). The failure of (CST) axons to re-enter the distal spinal host tissue maybe in part related to the presence of the so-called glial scar including the chondroitin sulfate proteoglycans (CSPGs), which inhibit axon growth (Fawcett and Asher 1999; Silver and Miller 2004). Neutralizing these inhibitors with blocking antibodies and modulating their downstream intracellular signaling (Hannila and Filbin 2008) or enzymatic degradation of the CSPGs resulted in improved axon regeneration (Kwok et al. 2008), including CST fibers (Iseda et al. 2008). CSPGs are more heavily expressed at the distal than the proximal interface after a complete spinal transection and this in itself makes re-entry of injured fibers into the distal cord much more complicated than the enhancement of the sprouting in areas proximal to the lesion (Bunge 2001). Here also, the different interaction or invasion of grafted cells (for instance, SC) embedded in a Matrigel matrix and the proximal versus the distal stumps of the spinal cord needs further investigation.

##### In summary

The implantation of Matrigel alone into the injured spinal cord does not stimulate regeneration of injured axons. Hence, Matrigel is used as a vehicle for the transplantation of cells/factors into the lesion gap after a spinal cord injury. Many experiments have been designed in which Matrigel is not only used as a vehicle to introduce SCs and stem cells into the lesion but also for local delivery molecules, which enhance the neurite growth like NT-3 and the formation of blood vessels like VEGF. Then, an enhanced ingrowth of anterogradely labeled CST fibers into the Matricel (+transplanted cells or molecules) is noted but at the same time there is an absence of re-entry of the CST fibers into the host in areas distal to the transplant. The latter maybe in part related to the presence of CSPGs, which inhibit axon growth.

#### Fibrin and fibronectin (mats)

**Fig. 3:**
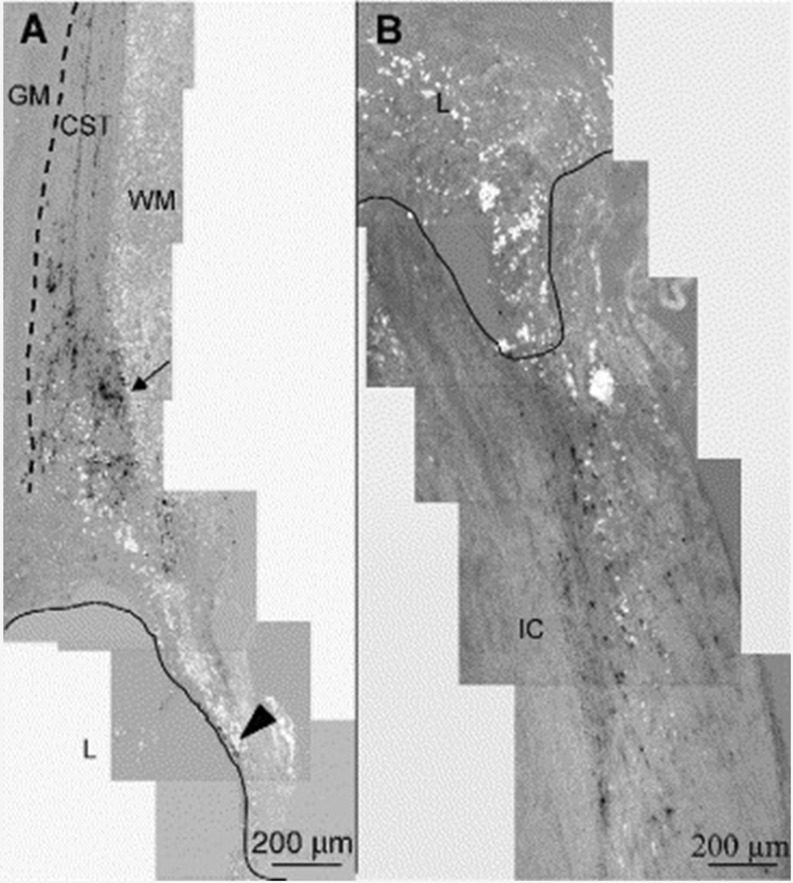
Corticospinal and ascending sensory tract tracing at 12 weeks. Parasagittal sections, with rostral cord oriented toward top, ventral surface toward left. **a** Biotin dextran amine (BDA) anterograde tracing of the corticospinal tract (*cst*) in a F-DS-NT-3 (1,000 ng/mL) treated cord. The CST extends from the rostral intact cord toward the lesion site. The CST is located just dorsal to the gray matter (*gm*, indicated by *dotted line*), in the most ventral part in the white matter (*wm*) of the dorsal funiculus. In all groups, as fibers approached the lesion (*l*, border indicated by *line*), the fibers were truncated or diverted to more dorsal white matter (*arrow*). Infrequently, CST-positive fibers were seen in the spared dorsal matter encircling the rostral lesion cavity (*arrowhead*). **b** Tracing of ascending sensory neurons with cholera toxin B (CTB) in F-DS-NT-3 (1,000 ng/mL) treated cord. In all groups, CTB staining ended abruptly at the lesion border with the intact cord (*lc*). Adapted from Taylor and Sakiyama-Elbert (2006), with permission

As already discussed (see “Matrigel”), the additional use of fibrin scaffolding is needed in order to stimulate the regrowth of injured CST fibers after use and in combination with various transplantation paradigms in the lesioned rat spinal cord (Guest et al. 1997a). Furthermore, fibronectin mats or fibrin scaffolds in themselves can be used to deliver neurotrophins (or growth factors) in a controlled manner and at the same time act as a physical bridge for regeneration. Fibrin-based tissue engineering scaffolds not only enhance neural fiber sprouting but at the same time delay the accumulation of reactive astrocytes at the lesion in a subacute model of spinal cord injury (Johnson et al. 2010). For optimal delivery of drugs into the lesioned spinal cord, it is required that the delivery system (DS) sequesters and protects the protein (or growth factor) until the appropriate time of release and, furthermore, that the release allows the drug to be available to regenerating neurons or axons over a longer period of time. An affinity-based delivery system based on fibrin scaffolds has been developed to provide sustained release of neurotrophins (Wood et al. 2010). Affinity-based delivery systems allow the release of growth factors to be controlled related to the degradation of the delivery system and surrounding fibrin matrix (Sakiyama-Elbert and Hubbell 2000). The delivery of NGF and GDNF from fibrin matrices containing the affinity-based delivery system has been shown to promote peripheral nerve regeneration in short-term in vivo studies and in particular the GDNF DS demonstrated superior functional recovery as compared to autograft controls (Wood et al. 2009, 2010). Also, an affinity-based delivery system to release NT-3 in a controlled manner from fibrin gels has been developed (Taylor et al. 2004): NT-3 was immobilized within fibrin gels via non-covalent interactions in order to slow the diffusion release. Then, the release of NT-3 is mediated through the cell-activated degradation of fibrin (Taylor et al. 2004). The immediate implantation of NT-3 containing fibrin scaffolds into the lesioned spinal cord enhanced the initial regenerative response (9 days after the lesion) by increasing neuronal fiber sprouting and cell migration into the lesion. This cellular response was, however, not accompanied by functional improvements (Taylor et al. 2006). In a follow-up study, the effect of controlled delivery of NT-3 from fibrin scaffolds acutely after spinal cord injury was evaluated at the chronic stages. At 12 weeks after injury and treatment, the animals treated with NT-3 fibrin scaffolds did not show functional improvements and, despite the fact that neuronal fibers were present inside the lesion, anterogradely traced CST fibers (where these fibers were truncated or diverted to the more dorsal white matter; Fig. 3; Taylor and Sakiyama-Elbert 2006) and dorsal sensory tract axons did not regrow into the lesion (Taylor and Sakiyama-Elbert 2006). In this context, a major disadvantage of the fibronectin mats as well as fibrin glue acting as bridge implants is their relatively rapid (1–2 weeks) degradation. Hence, an alternative approach would be to perform this treatment in the chronic phase after the lesion has stabilized.

##### In summary

Despite significant improvements in design of fibrin gels/mats and controlled delivery of neurotrophins (like NT-3), a major disadvantage of the fibronectin mats and fibrin gels is their relatively rapid degradation. Hence, timing of the implantation of (the presently available) fibronectin/fibrin bridges into the lesioned spinal cord is important; timed implantation at chronic stages may result in significant regeneration of injured CNS fibers including the CST.

#### Poly (alpha-hydroxy acids)

The aliphatic polyesters derived from lactide, glycolide and E-caprolactone are completely resorbable and biocompatible in the central nervous system (Menei et al. 1993: Gautier et al. 1998). Poly(alpha-hydroxy acids), which include poly(lactic acid) (PLA), poly(glycolic) and poly(lactic-co-glycolic acid) (PLGA) are materials well investigated for tissue repair and cell transplantation (Fournier et al. 2003). The resorption rate and the mechanical properties of aliphatic polyesters can be adjusted; as they depend primarily on the ratio of L-Lactic acid, D-Lactic acid, glycolic acid; and E-caprolactone repeating units. In particular; the PLGA oligomers and their breakdown products have no adverse effects on the morphology, survival and proliferation of cultured Schwann cells (Gautier et al. 1998). In vivo cylinders made of PLGA were integrated into the spinal tissue 2 weeks after implantation, unlike cylinders made of PLA. At all time points after surgery, the glial and inflammatory reaction near the lesion site was unaffected by the poly-(alpha-hydroxyacids) and growth-associated protein 43 (GAP-43) indicative for regenerating axons was observed in fibers in the vicinity of the injury site and the remains of PLGA and PLA cylinders (Gautier et al. 1998).

Three-dimensional PLGA or PLA scaffolds can easily be used for the introduction of therapeutic cells into the spinal lesion site. A type of PLGA nerve conduits is fabricated with controllable aligned multiple channels and a hierarchical pore microstructure in the wall (He et al. 2009). It then emerged that these PLGA conduits formed a favorable environment for the adhesion of bone marrow-derived mesenchymal stem cells (MSCs) and Schwann cells (SC). For instance, PLGA scaffold–Schwann cell constructs improved hindlimb motor recovery in a completely transected spinal cord model (Zhang et al. 2007). Retrograde tract tracing analysis (Fast Blue injections distal to the lesion and PLGA–SC scaffold) revealed neuronal labeling of pyramidal cells in the cortex, known to be the cells of origin of the CST (Chen et al. 2009). The use and implantation of neural stem cells (NSC), integrated into a PLGA scaffold placed into the injured rat spinal cord, promoted long-term improvement of function (Teng et al. 2002). Furthermore, anterograde tract tracing, at 70 days post-injury, demonstrated CST fibers passing through the injury epicenter to the caudal cord. Although not quantified, the authors report that the regenerating CST axons in and beyond the PLGA–NSC scaffolds are characterized by their tortuous path, a phenomenon that is most consistent with morphological profiles generally seen in regenerating axons as opposed to spared fibers, which retain their straight and non-tortuous profile (Teng et al. 2002).

Another use and development of a PLGA-based neural construct was developed by Zhang et al. (2007): NT-3 and trkC gene-modified neural stem cells were seeded in PLGA, thereby creating an artificial construct that permitted NSCs to differentiate into neurons establishing connections with each other and exhibiting synaptic activity (Zhang et al. 2012; Xiong et al. 2009). However, with the use of this type of construct, no anterogradely labeled fibers crossing the injury site were noted, suggesting the limited ability of CST axonal regeneration (Du et al. 2011).

**Fig. 4:**
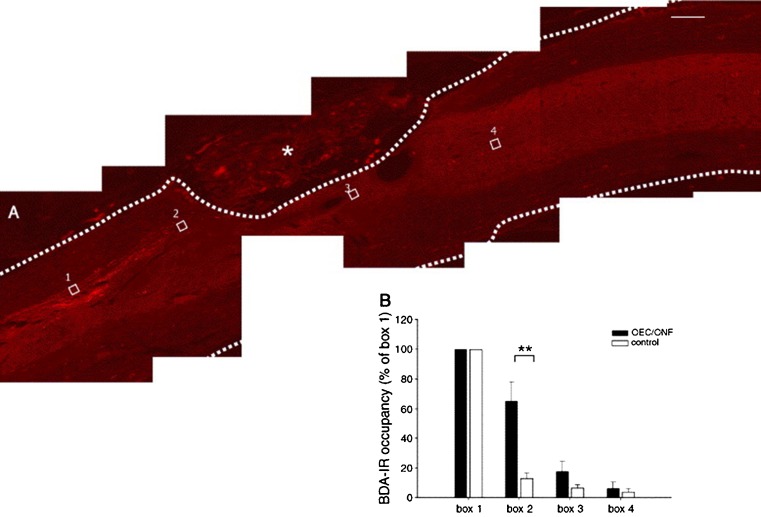
Biotinylated dextran amine (BDA)-labeled corticospinal tract axons and response to OEC/ONF intervention. BDA-labeled corticospinal axons were observed to approach the graft/lesion site, but only very few grew underneath the lesion and into the caudal host spinal cord. No labeled corticospinal axons penetrated the graft/lesion site. **a** Sagittal spinal cord section of an OEC/ONF-transplanted animal. The* dotted line* represents the delineation of the spinal cord; the OEC/ONF–biomatrix complex is visible within the lesion site. The* four boxes* represent the boxes used in the quantitative analysis. **b** A significantly higher BDA-labeling is present directly rostral to the injury site (*box 2*) in transplanted animals versus control animals.* Scale bar* 200 um. Adapted from Deumens et al. (2006), with permission

Also, PLA matrices have shown biocompatibility with cultured cells including glial cells (Maquet et al. 2000). Whereas PLA matrices consist in macroporous polylactide foams, which are highly oriented (Maquet et al. 2000), the transplantation of these matrices into the injured spinal cord resulted in preferential axon growth along the main pore direction (Blacher et al. 2003). In vitro analysis of aligned glial/biomatrix (PLA) complexes demonstrated that this resulted in directing the neurite growth of neurons cultured on top of these matrices but at the same time did not enhance the neurite growth (Deumens et al. 2004). In particular, OECs have been demonstrated to be very potent cells in stimulating regrowth of injured CST fibers, albeit only in very small spinal cord lesions (Li et al. 1997). Hence, a strategy was developed that used aligned 2-dimensional PLA bridges and OEC and olfactory nerve fibroblast (ONFs) cells transplanted into large spinal lesion gaps (Deumens et al. 2006). In order to further optimize this strategy, two OEC/ONF injections were intraspinally placed either at 1 mm rostral or 1 mm caudal to the lesion gap, thereby creating an OEC/ONF continuum. Anatomically, this approach resulted in an enhanced presence of CST axons directly rostral to the lesion gap but semi-quantitative analysis revealed no regrowth of CST axons through the lesion site (Fig. 4) (Deumens et al. 2006). Although small functional behavioral improvements in the rat were noted using the OEC/ONF-PLA continuum strategy, it was concluded that this multifactorial transplantation strategy has limited effects on repair of large spinal lesion gaps. Two critical remarks need to be made, however, before we may conclude that OEC/ONF–biomatrix bridges are not succesful in repair of injured spinal cord. First, the bridge used consisted in a 2-D PLA biomatrix where a 3-D biomatrix may better mimick the developmental situation of the outgrowing CST and therefore potentially result in better regrowth. Second, the OEC/ONF cells did not survive on the PLA matrices and thus no continuum was formed. The breakdown of the PLA may have caused a small increase in toxic substances in the lesion area and, in combination with the putative high concentrations of toxic substances released by pathological processes into the large lesion gaps, may account for OEC/ONF cell death and finally the absence of regrowing CST fibers. A similar observation (toxicity and cell death) was made in Schwann cells genetically modified to secrete a bi-functional neurotrophin and seeded on PLA macroporous guidance scaffolds in the lesioned spinal cord (Hurtado et al. 2006). From these observations, it makes sense to use PLGA guidance scaffolds seeded with glial cells. PLGA oligomers and their breakdown products have been reported to have no effects on the morphology, survival and proliferation of cultured Schwann cells (Gautier et al. 1998). In this respect and in view of the important role that vascularization (see also “Matrigel”) may play in optimalization of (CST) regrowth, PLGA degradable matrices seeded with a co-culture of neural progenitor and endothelial cells were developed. Here, an increase of blood vessel density was observed and formation of a blood–spinal cord barrier following SCI (Rauch et al. 2009). Unfortunately, analysis of CST was not included into this study (Rauch et al. 2009).

PLGA does not only function as scaffolds for cell polymers but can be used for controlled release drug delivery: neurotrophic factors can be encapsulated into PLGA nanoparticles injected into the injured spinal cord and used for a controlled and sustained release (Wang et al. 2006; Astete and Sabliov 2006; Benoit et al. 2000). For instance, the therapeutic effect of PLGA nanoparticles encapsulating glial-derived neurotrophic factor (GDNF), intraspinally injected into the acutely injured spinal cord (Wang et al. 2006), was evaluated by examination of the preservation of neurofilament (NF)-stained fibers and functional recovery using the BBB locomotor rating scale (Basso et al. 1995). In vitro and in vivo analysis showed a continuous release of GDNF from the PLGA nanoparticles and that this induced an increase in neuronal survival and improves better hindlimb motor function in SCI rats (Wang et al. 2006). Interestingly, the PLGA-GDNF nanoparticles had no effect on gliosis when compared to that observed in SCI rats receiving PLGA injection (Wang et al. 2006).

##### In summary

Biodegradation and breakdown of PLA may result in an increase in toxic substances in the lesion followed by cell death, including the transplanted cells. Future design of three-dimensional PLGA guidance scaffolds seeded with glial cells (particularly promising are the OEC/ONF) with the main aim to re-create a continuum for regrowth of injured CST fibers may result in significant ingrowth into the bridge and re-entry of injured CST fibers into the host. The use of PLGA nanoparticles for a controlled and sustained release of neurotrophins is a very promising development in the field of biomatrices and bridging the injured spinal cord and has already resulted in improved hindlimb motor function in SCI rats. Follow-up studies are needed to demonstrate the effect of local delivery of the PLGA nanoparticles encapsulating neurotrophins on CST regrowth.

#### Polyethylene glycol and chitosan

Polyethylene glycol (PEG) is a highly water soluble polymer and has served as a therapeutic agent to reconstruct the phospholipid bilayers of damaged cell membranes (or fusogenic activity). Based on this fusogenic property, the systemic intravenous application of 30% PEG in rats that underwent 35-g clip compression at cervical eight (C8) resulted in a reduced neurofilament degradation and apoptotic cell death in the lesion area finally resulting in a modest neurobehavioral recovery after SCI (Baptiste et al. 2009). Retrograde tract tracing studies were not focused at the cortex (and CST) but an increase in labeled reticulospinal cells was noted (Baptiste et al. 2009). Hence, PEG protects key axonal cytoskeletal proteins after SCI and this protection is associated with axonal preservation (Cho and Borgens 2012). Also, the local application and intraspinal single injection of PEG immediately after or at 7 h after spinal cord injury was found to reduce cystic volume and to increase the volume of remaining intact spinal cord parenchyma (Borgens et al 2002; Duerstock and Borgens 2002; Novikova et al. 2003). In a pilot trial, the intravenous application of PEG within 72 h after admission of neurologically complete cases of paraplegia in dogs was shown to result in significant neurological recovery of the PEG-treated animals as compared to the untreated controls over a 6- to 8-week trial period (Laverty et al. 2004). The neurological improvements included deep and voluntary pain perception, conscious perception of the hindlimbs as well as electrophysiological recording of the somatosensory evoked potentials, whereas at the same time no clinically relevant adverse effects to the polymer injection were noted (Laverty et al. 2004).

In the context of membrane resealing, not only is PEG important but also another striking example has been demonstrated recently by using chitosan (Cho et al. 2010a, b). Chitosan is bio-compatible, biodegradable, non-toxic and can easily be prepared from the exoskeletons of crustaceans. Chitosan has widely been used as a drug carrier and wound healer and first indications are that this natural polymer after topical application is capable of inducing the sealing of neuronal membranes and restores the conduction of nerve impulses through the length of the spinal cord preferentially targeting the region of damage (Cho et al. 2010a, b). In addition, chitosan can easily participate in the preparation of microcapsules or micro/nano-spheres serving as a carrier, which is particularly suitable for controlled drug release (for review, see Cho and Borgens 2012)

In addition to its fusogenic capability and reconstruction of injured axonal membranes in SCI, PEG is used to increase the efficacy of intrathecal protein administration into the injured SCI. PEG is the most widely used polymer for covalent attachment of synthetic polymers to proteins and has been shown to improve protein half-life and penetration into tissues (Roberts et al. 2002). PEGylation of neurotrophic molecules like BDNF (Soderquist et al. 2009) and fibroblast growth factor-2 (FGF-2) (Kang et al. 2010) has recently been described. In both examples, the PEGylation resulted in an improvement in biological activity of the protein, improvement of the half-life (after intrathecal or delivery) and at the same time an enhanced tissue penetration of the neurotropins (Soderquist et al. 2009; Kang et al. 2010). Futher research is needed to demonstrate a possible positive effect of intrathecal delivery and PEGylation of neurotrophins and on CST regrowth.

In view of the use of PEG in SCI and repair, two important recent development needs to be mentioned here. First, the functionalization of single-walled carbon nanotubes with PEG (SWNT-PEG): the injection of SWNT-PEG into the lesion at T9 1 week after a complete transection decreased the lesion volume, did not increase reactive gliosis and at the same time increased the number of neurofilament and anterogradely labeled CST fibers in the lesion (Roman et al. 2011). It should be noted that, although SWNT-PEG resulted in a significant increase in CST fibers, the absolute numbers are still extremely low, varying between 1 and 3 fibers in the rostral-epicenter region (Roman et al. 2011). Second, the development of injectable hydrogel scaffolds based on PEG lightly cross-linked with poly (N-isopropylacrylamide) (PNIPAAm) for local delivery of neurotrophins like BDNF or NT-3 into the lesion gap after SCI (Conova et al. 2011; Comolli et al. 2009): it was observed that the release of BDNF and NT-3 was sustained for up to 4 weeks and that the bioactivity of these neurotrophins was still very good (Comolli et a l. 2009). The PNIP-AAm-PEG hydrogels were also compatible with bone marrow stromal cells, allowing their survival and attachment for up to 31 days (Comolli et al. 2009).

##### In summary

Based on the membrane resealing capacity of PEG (and chitosan), the intravenous application has been shown to result in axonal preservation and a significant neurological recovery after spinal cord injury. PEGylation of neurotrophins, functionalization of carbon nanotubes with PEG, or the development of injectable hydrogel scaffolds based on PEG are interesting and important improvements in design of successful biomatrices and bridges for repair of injured spinal cord, including the CST.

## Discussion

The use of degradable biomatrices for implantation into the lesioned mammalian spinal cord has led to interesting and important findings on CNS regeneration in general and also in view of CST regrowth. As can be deduced from the approaches chosen, the implementation of factors and or cells important during the development of the spinal cord and combined and/or integrated into a biodegradable matrix often formed the fundaments of repair strategies. With respect to the injured CST, main efforts have been taken to bridge the lesion based on the re-creation or reconstruction of a 3-D alignment of outgrowth promoting cells (immature astroglial cells), as this typical structure is known to be present and important during guidance and development of this tract. Furthermore, bridging the lesion and stimulation of the regrowth of injured CST fibers is often triggered by application of the neurotrophin-3 (NT-3), a molecule also known to be important during the development of this tract.

In general, it can be concluded that various approaches have led to a significant ingrowth of anterogradely labeled regrowing CST into the bridges. It is difficult to compare the quality of the various bridges used but at the present moment not one single approach stands out. Furthermore, the major drawback of all bridges currently used and developed is the fact that no re-entry of CST fibers from the graft into the host tissue is noted. It is here where most research should be focused at. Despite the fact that various degradable biomaterials in themselves do not enhance the formation of scar tissue or even minimize the impermissiveness of the host tissue, the CST axons do not re-enter; obviously, the CST fibers like it too much in the graft and are not triggered (enough) to re-enter the host. Here, various aspects, based on our developmental know-how, might be directive and inform us about which approach should be taken in future design of CNS bridges and CST regrowth. The presence of neurotrop(h)ic molecules but also the 3D alignment of the glial cells during development, is restricted in time and only needed during the various phases of CST outgrowth. In future designs of bridges and biomaterials, mimicking a developmental CST environment is of the utmost importance; not only the presence of the cue needed for regrowth but equally important is the disappearance or decrease in concentration of the guidance molecule after some time. If we again use the developmental CST as the most optimal model needed for regrowth of injured CST fibers in the adult mammalian spinal cord, we have learned that the development of the CST tract is characterized by cellular interactions, which are changing during the spinal outgrowth in place and in time. It has been shown, for instance, that for spinal target innervation the most common mechanism used is collateral formation or back-branching, a phenomenon, at least to my knowledge that has never been observed (or studied) in regrowing injured CST fibers. This back-branching occurs after a waiting period of several days at a defined spinal level and the neurotrophin NT-3 might be important, although the precise mechanism underlying the formation of the CST-collaterals is not yet known. Whereas the injured CST axons should be triggered to re-enter the host tissue, it should be stressed that the failure of (CST) axons to re-enter the distal spinal host tissue maybe in part related to the presence of the so-called glial scar, including the chondroitin sulfate proteoglycans (CSPGs), which inhibit axon growth (Fawcett and Asher 1999; Silver and Miller 2004). The much more intense expression of CSPGs at the distal as compared to the proximal interface after a complete spinal transection may account for the absence of regrowth of CST fibers (Bunge 2001). CST axons are known to terminate in CSPG-rich regions after spinal hemisection or contusion in rats (Iseda et al. 2008). Hence, treatment with chondroitinase ABC and the enzymatic degradation of the CSPGs resulted in the re-entry of a small amount of the anterogradely labeled CST fibers into host tissue (for 0.5 mm!!) in the hemisected spinal cord but not in the contused spinal cord (Iseda et al. 2008). Obviously, in spinal cord contusion, other axonal growth inhibitors persist in the gliotic areas as compared to the hemisected cord.

### Future: suggestions and considerations

Future development of smart biodegradable bridges for CST regrowth in the injured spinal cord should focus on timed release of factors and molecules required for CST regrowth, with a very special emphasis at the re-entry of the corticospinal fibers into the host-tissue. The complex of biodegradability of the matrix, the bio-availability of the growth-stimulating molecule and the three dimensional structure of the graft should result in an *ON* (“ingrowth of CST fibers into the bridge”) and (maybe even more important!!) an *OFF* mode (“allowing re-entry of the CST fibers out of the bridge into the caudal host”). In this, the use of a bridge does not stand alone: the formation of a continuum based on outgrowth promoting capacities not only related to the bridge but also including the rostral and caudal host tissue (as initially designed and described for PLA-bridges and OEC cells; Deumens et al. 2006) is almost certainly a pre-requisite for succesful regeneration of the injured CST.

In the “Introduction”, it was stated that the corticospinal tract is considered the ultimate challenge to demonstrate whether a repair strategy is succesful in regeneration of the injured mammalian spinal cord. In view of the almost unlimited number of attempts and approaches undertaken (of which in this review only the biodegradable bridges have been discussed!!) to achieve this goal and the still very limited success, one might suggest that the focus should no longer be on this tract. However, it is of eminent importance to understand the regeneration requirements of each individual tract in the repair of the injured spinal cord. As can be deduced from most approaches taken, evaluation of the success of the bridge/material chosen starts with the effect of the (new) bridging therapy on sprouting in general. This often results in promising effects on the (neurofilament-immunoreactive) regrowth of a variety of CNS fiber populations. However, is this what we want? Obviously not, because, if for instance the induction of regrowth of injured motor fibers (corticospinal/rubrospinal) into the graft occurs and at the same time an unintended induction of sprouting of sensory fibers happens, this eventually results in deleterious consequences. The indiscriminate promotion of sprouting of fibers within the spinal cord after injury will result in the formation of new and inappropriate connections between primary afferent fibers, conveying both noxious and non-noxious sensory information onto hyperexcitable dorsal horn nociceptive neurons. A striking example in this respect is the use of OEC transplantation, which not only resulted in sprouting of motor fiber regrowth but also evoked a sprouting response of the pain afferents in the tissue surrounding the spinal lesion (Richter and Roskams 2008). In these OEC-treated animals, autotomy of the hindlimbs was observed, which is regarded as a behavioral sign of spontaneous pain (Kauppila 1998). From this point of view, the behavioral analysis of repair strategies like those described in this review, based on the induction of sprouting of injured CNS (and CST) fibers in the lesioned spinal cord, should include the use of (chronic) pain assays. Furthermore, this example illustrates that the indiscriminate promotion of sprouting after SCI is not the correct way but that selective regrowth of identified fiber tracts is needed. Or, at least as a minimum, the new bridging therapy should balance between restoration of motor function and at the same time not induce primary afferent fiber sprouting that can contribute to chronic pain. Recent advances in our understanding of molecular inhibitors and promoters of selective sprouting of tract- and class-specific fibers have been reviewed (Deumens et al. 2008)

Also, from a (selective) point of motor restoration, the regeneration of individual fiber systems is needed in order to develop an optimal repair strategy and motor recovery. In this respect, one important question needs to be addressed: what is the relationship between an anatomically observed response related to the function repair and recovery of function? Or, do we need all injured (CST) fibers to regenerate?

With respect to the CST, the question of which behavioral deficits are due to CST lesioning in adult rats is still not fully answered. Although the transection of the CST in the rat leads to, for instance, a loss of contact placing, the role of this tract in the control of distal and proximal limb movements is still not fully understood. The good news is that it has been documented that a very low percentage of regrowing fibers at the anatomical level may account for a complete recovery of the placing reflex (Bregman et al. 1995). These observations have led to the conclusion that recovery of specific functions can be mediated by small numbers of descending (CST) fibers, which then influence the local spinal circuitry. This gives hope for the future.
